# Efficacy of a novel topical combination of esafoxolaner, eprinomectin and praziquantel against adult cat flea *Ctenocephalides felis* and flea egg production in cats

**DOI:** 10.1051/parasite/2021017

**Published:** 2021-04-02

**Authors:** Eric Tielemans, Prescillia Buellet, David Young, Alta Viljoen, Julian Liebenberg, Joe Prullage

**Affiliations:** 1 Boehringer-Ingelheim Animal Health 29 Avenue Tony Garnier 69007 Lyon France; 2 Boehringer-Ingelheim Animal Health, Missouri Research Center 6498 Jade Rd. Fulton MO 65251 USA; 3 Young Veterinary Research Services 7243 East Avenue Turlock CA 95380-9124 USA; 4 Clinvet International (Pty) Ltd. P.O. Box 11186, Universitas Bloemfontein 9321 Republic of South Africa

**Keywords:** Cat, *Ctenocephalides felis*, Esafoxolaner, Fleas, Eggs, Efficacy

## Abstract

Esafoxolaner, a purified enantiomer of afoxolaner with insecticidal and acaricidal properties, is combined with eprinomectin and praziquantel in NexGard^®^ Combo, a novel topical endectoparasiticide formulation for cats. The efficacy of this novel formulation against adult and immature stages of *Ctenocephalides felis* fleas was tested in four experimental studies. Two studies were designed to test adulticide efficacy, one to test inhibition of immature stages, and one to test both adulticide efficacy and inhibition of immature stages. In each study, cats were randomly allocated to a placebo control group or to a novel formulation group treated once at the minimum recommended dose. Cats were experimentally infested weekly for one to two months with unfed *C. felis* originating from North America or Europe. For adulticide efficacy evaluations, live fleas were counted 24 h after treatment and after subsequent weekly infestations. For immature stages, flea eggs were collected and counted weekly for evaluation of egg production inhibition and incubated for larval hatching evaluation. In the three studies testing adult fleas, curative efficacies, 24 h after treatment, were 92.1%, 98.3% and 99.7%; preventive weekly efficacies, 24 h after weekly infestations, remained higher than 95.5% for at least one month. In the two studies testing immature stages, egg production and larval hatching was significantly reduced for at least one month. These studies provide robust evidence of efficacy of the novel formulation against experimental adult flea infestations and for the prevention of environmental contamination by immature flea stages, for at least one month.

## Abstract (fr)

L’esafoxolaner, un énantiomère purifié de l’afoxolaner aux propriétés insecticides et acaricides, est associé à l’éprinomectine et au praziquantel dans NexGard^®^ Combo, une nouvelle formulation endectoparasiticide topique pour chats. L’efficacité de cette nouvelle formulation contre les stades adultes et immatures des puces *Ctenocephalides felis* a été testée dans quatre études expérimentales. Deux études ont été conçues pour tester l’efficacité des adulticides, une pour tester l’inhibition des stades immatures et une pour tester à la fois l’efficacité des adulticides et l’inhibition des stades immatures. Dans chaque étude, les chats ont été répartis au hasard dans un groupe témoin placebo ou dans un groupe de formulation traité une fois par la nouvelle formulation à la dose minimale recommandée. Des chats ont été expérimentalement infestés chaque semaine pendant un à deux mois par des *C. felis* non nourris provenant d’Amérique du Nord ou d’Europe. Pour les évaluations de l’efficacité des adulticides, les puces vivantes ont été comptées 24 heures après le traitement et après les infestations hebdomadaires suivantes. Pour les stades immatures, les œufs de puces ont été collectés et comptés chaque semaine pour l’évaluation de l’inhibition de la production d’œufs, et incubés pour l’évaluation de l’éclosion des larves. Dans les trois études testant les puces adultes, les efficacités curatives, 24 heures après le traitement, étaient de 92,1 %, 98,3 % et 99,7 %, et les efficacités hebdomadaires préventives, 24 heures après les infestations hebdomadaires, sont restées supérieures à 95,5 % pendant au moins un mois. Dans les deux études testant les stades immatures, la production d’œufs et l’éclosion des larves ont été considérablement réduites pendant au moins un mois. Ces études fournissent des preuves solides de l’efficacité de la nouvelle formulation contre les infestations expérimentales de puces adultes et pour la prévention de la contamination environnementale par les stades de puces immatures, pendant au moins un mois.

## Introduction

Some of the most frequent parasitic disorders of felines are caused by fleas, major ectoparasites of carnivores and other mammalian species worldwide [8, 20, 21, 23]. *Ctenocephalides felis* is the main flea species found in both cats and dogs globally. The species is not fully host-specific and it can infest or take blood meals from other mammals such as wild canids and felids, ruminants, rodents or humans [8, 9, 20, 21]. Flea infestations represent a significant veterinary and public health concern. Flea bites induce discomfort and skin inflammatory reactions that can result in dermatological signs such as alopecia, erythema, or moist dermatitis, and systemic disorders such as anemia [8, 22, 23]. Flea bite hypersensitivity, also called flea allergy dermatitis (FAD), is one of the most common dermatological conditions in companion animals and includes signs such as pruritus, crusts, alopecia, and miliary dermatitis [11, 22]. The cat flea can also transmit zoonotic diseases such as *Rickettsia felis*, the agent of flea-borne spotted fever, and *Bartonella henselae*, the agent of cat scratch disease [1–4, 9, 13, 16, 18]. Additionally, fleas are the intermediate host for *Dipylidium caninum* [7, 10], a common cestode of cats and dogs.

Pet owners often treat their animal only when they see fleas or flea infestation-related symptoms, but this approach cannot break the epidemiological cycle as adult fleas infesting animals represent a minor part of the total flea population [14, 23]. In an infested environment, the vast majority of the flea population is composed of eggs, larvae and pupae. Thus, sustained efficacy against new infestations is important for an optimal flea control program, as well as the inhibition of flea egg production and/or development.

NexGard^®^ Combo, a novel topical combination of esafoxolaner, eprinomectin and praziquantel has been designed to offer a wide spectrum of antiparasitic activity.

The objectives of the four studies presented here were to assess the efficacy of this novel formulation for the treatment and control of adult *C. felis* and for the inhibition of flea egg production and larval development, when administered topically to cats.

## Materials and methods

### Ethics

The study protocols were reviewed and approved by the Sponsor’s and local Institutional Animal Care and Use Committees. Cats were managed and handled with due regard for their wellbeing.

### Study designs

The four studies were designed in accordance with the “World Association for the Advancement of Veterinary Parasitology (W.A.A.V.P.) guidelines for evaluating the efficacy of parasiticides for the treatment, prevention and control of flea and tick infestation on dogs and cats” [19], and in accordance with Good Clinical Practices as described in “International Cooperation on Harmonization of Technical Requirements for Registration of Veterinary Medicinal Products (VICH) guideline GL9”.

The four studies had different objectives, contexts and designs, as summarized in Tables 1 and 2. The studies were conducted in three licensed laboratories, in South Africa (two studies), in France, and in California, United States. Overall, the objectives were to measure adulticide efficacy and egg production inhibition, for at least one month, of one application of the novel formulation at the minimum recommended dose against flea isolates of European or American origin.

**Table 1:** Study objectives and contexts.

	Objective(s)	Location/date	*C. felis* strain origin	Variable used for randomization	Variable(s) of efficacy
Study #1	Adulticide efficacy for 8 weeks	France/Jan–Mar 2018	Germany	Pre-treatment adult flea count 24 h	24-h weekly flea reduction
Study #2	Adulticide efficacy and egg production for 1 month	USA (CA)/Aug–Sep 2017	California	Pre-treatment adult flea count 24 h	24-h weekly flea reduction; egg production and larval hatching reduction
Study #3	Inhibition of egg production for 1 month	South Africa/Oct–Nov 2017	Hungary	Pre-treatment larval hatching	Egg production and larval hatching reduction
Study #4	Adulticide efficacy for 5 weeks	South Africa/Nov–Dec 2019	Germany	Pre-treatment adult flea count 24 h	24-h weekly flea reduction

**Table 2:** Study designs.

	Treatment^1^	Infestations (~100 unfed adult *C. felis*)	Flea counts (comb count)	Egg collection^2^	Egg incubation^3^
Study #1	Day 0	Days −1, 6, 13, 20, 27, 34, 41, 48, 55	Days 1, 7, 14, 21, 28, 35, 42, 49, 56	NA	NA
Study #2	Day 0	Days −1, 7, 14, 21, 30	Days 1, 8, 15, 22, 31	Days 0–1, 7–8, 14–15, 21–22, 30–31	Days 0–4, 7–11, 14–18, 21–25, 30–34
Study #3	Day 0	Days 1, 8, 15, 22, 29	Days 3, 10, 17, 24, 31 (removal only)	Days 1–3, 8–10, 15–17, 22–24, 29–31	Days 1–6, 8–13, 15–20, 22–27, 29–34
Study #4	Day 0	Days −1, 6, 13, 20, 30, 37	Days 1, 7, 14, 21, 31, 38	NA	NA

1 Cat were treated once topically with Nexgard^®^ Combo at the minimum recommended dose (the control group was treated with a placebo).

2 Cats were placed in individual enclosures for egg collection; eggs were collected daily and incubated on the day of collection.

3 Larval hatching assessment performed on the last day of incubation.

Each study was conducted using a randomized design, with cats allocated to either a placebo control or a novel formulation treated group, with blocking based on pre-treatment flea infestation or larval hatching levels.

The efficacy assessments against adult fleas were based on comparison of adult flea counts in the control and treated group 24 h after treatment (curative efficacy), or 24 h after subsequent weekly infestations (preventive efficacy). The efficacy assessments against immature stages were based on two variables: egg production inhibition, i.e. comparison of number of eggs found in the control and treated group at identical weekly timepoints; and larval hatching inhibition, i.e. comparison of larvae hatched from collected eggs in the control and treated groups after incubation.

All personnel collecting animal health and efficacy data were blinded to treatment.

### Animals and housing

The cats were healthy, purpose-bred laboratory Domestic Short/Long-hair cats, belonging to the local colony of each laboratory, and are described in Table 3. Cats were single-housed (and placed on wire mesh flooring when individual egg collection was performed) at least during the periods of flea infestation and flea removal (up to 48 h).

**Table 3:** Animal details.

	Origin	Number and sex	Bodyweight (kg)	Age (months)
Study #1	France	9 males, 9 females	2.1–3.7	4.3–6.7
Study #2	USA	9 males, 11 females	2.8–7.8	16–101
Study #3	South Africa	10 males, 14 females	2.4–4.4	12–89
Study #4	South Africa	10 males, 10 females	2.7–4.6	12–24

### Flea strains

All fleas were laboratory-maintained *C. felis* colonies originally sourced from the field. The flea colonies were not known to be resistant to ectoparasiticide compounds and had not previously been subjected to ectoparasiticide challenge. Genetic enrichments of the colonies were performed at least every 5 years. The different flea isolates had an unknown specific pathogen-free status.

The isolate used in Study #1 originated from Germany and was maintained on an artificial membrane device. The isolate used in Study #2 originated from Stanislaus County, California, USA, and was maintained on cats. The isolate used in the two South African studies (Studies #3 and #4) originated from the field in Germany, and was maintained on cats.

### Treatment and health observations

Cats were treated once on Day 0. The treatment was applied on one spot directly on the skin, after parting the hair, in the midline of the neck between the base of the skull and the shoulder blades. Cats assigned to the placebo control group were treated with mineral oil at 0.12 mL/kg; cats assigned to the novel formulation group were treated at the minimum recommended dose of 0.12 mL/kg, delivering 1.44 mg/kg esafoxolaner, 0.48 mg/kg eprinomectin and 10.0 mg/kg praziquantel.

Health observations were conducted daily and at hourly intervals for 4 h after treatment in all studies to detect any treatment-related or unrelated health abnormality.

### Flea infestations and counts

Each cat was infested weekly with approximately 100 unfed adult *C. felis*. Fleas were removed and counted via thorough combing of all body areas with a fine-tooth flea comb. The schedule of flea infestation and count per study is detailed in Table 2.

### Flea eggs collection and larval hatch assessment

Flea eggs falling from each cat were collected from a pan under a wire mesh floor over a period of two days following infestation. Collected eggs were placed in individual containers for incubation at approximately 25 °C and 80% relative humidity. After 4–6 days of incubation, hatched flea larvae were counted.

### Statistical analysis

For the evaluation of efficacy against adult fleas, arithmetic means were calculated for each treatment group at each weekly timepoint. The percent efficacy was calculated as [(*C* − *T*)/*C*] × 100, where *C* was the arithmetic mean of the flea counts among the placebo-treated cats, and *T* was the arithmetic mean among the novel formulation-treated cats.

For the evaluation of efficacy against immature flea stages, geometric means of collected eggs were calculated for each treatment group at each weekly timepoint. The percent efficacy was calculated as [(*C* − *T*)/*C*] × 100, where *C* was the geometric mean of the flea counts among the placebo-treated cats, and *T* was the geometric mean among the novel formulation-treated cats.

For the evaluation of viability of eggs, the proportion of larval hatch from the total number of eggs collected was calculated at each timepoint. If both treatment groups at a given timepoint had more than two animals for which eggs were collected, the proportion of larval hatch of the novel formulation-treated group was compared to the proportion of larval hatch from the placebo-treated group.

For counts of adult fleas and eggs, the log-counts of the two groups were compared using the MIXED procedure in SAS Version 9.4 with treatment as a fixed effect and allocation block as a random effect. For the evaluation of viability of eggs, the proportions of larval hatches of the two groups were compared using the GLIMMIX procedure in SAS Version 9.4. For this analysis, block was included as a random effect, treatment group was the fixed effect, and the link function was the logit.

All treatment comparisons utilized a (two-sided) 5% significance level.

## Results

### Efficacy against adult fleas (Studies #1, #2 and #4)

The weekly mean flea counts and percent efficacy results obtained in the three studies are summarized in Table 4.

**Table 4:** *Ctenocephalides felis* adulticide efficacy.

	*n* cats	Arithmetic means (AM) of live fleas and % efficacy on indicated days									
Study #1			Day 1	Day 7	Day 14	Day 21	Day 28	Day 35	Day 42	Day 49	Day 56
Control group	9	AM	74.2	74.0	82.2	69.3	57.6	66.9	63.7	60.4	69.4
Treated group	9	AM	0.2	0.0	0.0	0.0	0.1	0.2	0.2	1.9	7.9
		% efficacy	99.7	100.0	100.0	100.0	99.8	99.7	99.7	96.9	88.6
Study #2			Day 1	Day 8	Day 15	Day 22	Day 31	–	–	–	–
Control group	10	AM	68.2	74.7	68.8	77.2	73.0	–	–	–	–
Treated group	10	AM	5.4	0.1	0.0	0.4	3.3	–	–	–	–
		% efficacy	92.1	99.9	100.0	99.5	95.5	–	–	–	–
Study #4			Day 1	Day 7	Day 14	Day 21	Day 31	Day 38	–	–	–
Control group	10	AM	64.5	69.6	69.5	72.1	62.4	49.2	–	–	–
Treated group	10	AM	1.1	0.0	0.3	0.3	0.7	4.8	–	–	–
		% efficacy	98.3	100.0	99.6	99.6	98.9	90.2	–	–	–

Note: *p*-values for comparison of the untreated and treated groups were <0.0001 at all timepoints.

The percent efficacy was calculated as [(*C* − *T*)/*C*] × 100, where *C* was the arithmetic mean of the flea counts among the placebo control cats, and T was the arithmetic mean among the Nexgard^®^ Combo-treated cats.

At all timepoints, the arithmetic means of live fleas in the control group were at least 49.2, confirming consistent robustness of the challenges in the three studies.

Curative efficacy on Day 1, 24 h after treatment, was 92.1%, 98.3% and 99.7% for each study, respectively. Preventive weekly efficacy, 24 h after weekly infestations, was at least 95.5% for one month in the three studies. One study was extended to eight weeks and at least 96.9% reduction was demonstrated for seven weeks; one study was extended to five weeks when the reduction was 90.2%. At all timepoints and in all studies, the number of live fleas was significantly lower on treated animals than on control animals (*p* < 0.0001).

### Flea egg production and larval hatch assessment (Studies #2 and #3)

The weekly mean egg counts, percent reduction of egg production, and larval hatching from collected eggs after incubation during the two studies are summarized in Table 5.

**Table 5:** *Ctenocephalides felis* egg production reduction and larval hatching inhibition.

	Flea egg production					Larval hatching proportion from collected eggs			
	Period of egg collection	GM^1^ control group	GM^1^ treated group	% reduction^2^	*p*-value	Period of incubation	% control group (*N*)	% treated group (*N*)	*p*-value
Study #2	Days 0–1	68.1	16.4	75.9	0.0128	Days 1–4	47 (10)	17.0 (9)	0.0058
*n* = 10	Days 7–8	35.6	0.1	99.8	<0.0001	Days 8–11	50 (9)	NA (1)	–*
	Days 14–15	28.2	0.0	100.0	<0.0001	Days 15–18	41 (10)	– (0)	–
	Days 21–22	38.7	0.0	100.0	<0.0001	Days 22–25	55 (9)	– (0)	–
	Days 30–31	36.5	0.0	100.0	<0.0001	Days 31–34	34 (10)	– (0)	–
Study #3	Days 1–3	138.6	9.5	93.2	0.0019	Days 3–6	68 (8)	26 (7)	0.0001
*n* = 8	Days 8–10	102.6	0.0	100.0	<0.0001	Days 10–13	84 (8)	– (0)	–
	Days 15–17	171.6	0.3	99.8	<0.0001	Days 17–20	73 (8)	NA (1)	–*
	Days 22–24	130.4	0.0	100.0	<0.0001	Days 24–27	68 (8)	– (0)	–
	Days 29–31	260.3	0.1	100.0	<0.0001	Days 31–34	72 (8)	NA (1)	–*

* Statistical analysis not performed as not meaningful because eggs produced on only one cat.

1 GM = Geometric mean; *n* = number of cats per group; *N* = number of cats from which egg(s) were collected and incubated.

2 The percent reduction was calculated as [(*C* − *T*)/*C*] × 100, where *C* was the geometric mean of the egg counts among the placebo control cats, and T was the geometric mean among the Nexgard^®^ Combo-treated cats.

In the first week after treatment, the reduction of egg production in Study #3 during Days 1 to 3 was 93.2% (*p* = 0.0019). In Study #2, a higher number of eggs was found in the treated group on Day 1 because in this study, the first flea infestation had been performed 24 h before treatment, on Day −1. Therefore, fleas were already producing eggs when cats were treated on Day 0 and continued to lay eggs until their death, resulting in lower egg reduction of 75.9%. From the second week after treatment to the end of the month, efficacy of the novel formulation to reduce production of *C. felis* eggs in both studies was at least ≥ 99.8%.

The proportion of larval hatching from collected eggs in the control groups in both studies ranged from 34% to 84%. Because of the low number of eggs in the treated groups, statistical analysis was only meaningful for the eggs collected and incubated on the week of treatment. Larval hatching from these eggs in the untreated control group and in the treated group was significantly different (*p* = 0.0058 in Study #2; *p* = 0.0001 in Study #3).

Results of the flea egg analyses indicate that the novel formulation was highly effective in reducing the number and the viability of flea eggs shed in the environment for at least one month in both studies.

No adverse reactions related to treatment were observed in any of the four studies.

## Discussion

The results of these four experimental studies illustrate the high level of efficacy of NexGard^®^ Combo for the reduction of adult flea infestations, and for the prevention of flea egg production. The three adulticide studies demonstrated that the new formulation is highly effective against existing flea infestations within 24 h of treatment and against subsequent infestations for at least one month, as previously reported for afoxolaner-based products used on dogs [6, 15].

The two studies targeting flea eggs demonstrated that the novel formulation causes a significant reduction in egg production, as previously found in dogs treated with afoxolaner [17]. This novel formulation quickly kills the fleas before they are able to lay eggs, which is necessary, in combination with the adulticide effect, to break the flea lifecycle at multiple stages [14, 23].

In a European survey on domestic cats examined in veterinary clinics for reasons unrelated to parasitic disorders, co-infestation with endo- and ectoparasites was observed in 14% of the subjects [5]. The control of multiple and various concurrent parasitic infestations by a range of cat parasites is important for cats but also public health [12, 24, 25].

This novel association of esafoxolaner, eprinomectin and praziquantel offers a broad spectrum of efficacy against the main parasites of cats including ecto- and endoparasites. Esafoxolaner is the purified and active (S)-enantiomer of afoxolaner, the racemic mixture. Afoxolaner has been proven effective against adult fleas and flea egg production in dogs [6, 15, 17]. In this novel formulation, the use of a purified enantiomer enables lowering of the dose and thus the potential for side effects, and interactions with the other active substances of the combination.

In addition to a high level of efficacy and safety, owner and cat compliance is an important feature for successful control of fleas. The simple conditions of use and of treatment application of this product should guarantee a high level of compliance.

This novel formulation provides pet owners and veterinarians with an effective solution for an integrated approach for cats presenting multiple parasitic infestations, or presenting risks of such parasitic infestations.

## Competing interest

The work reported herein was funded by Boehringer-Ingelheim. The authors are current employees of Boehringer-Ingelheim Animal Health or external organizations. Other than that, the authors declare no conflict of interest. This document is provided for scientific purposes only. Any reference to a brand or trademark herein is for information purposes only and is not intended for any commercial purposes or to dilute the rights of the respective owners of the brand(s) or trademark(s).

NexGard^®^ is a registered trademark of the Boehringer-Ingelheim Group.
